# Safety Evaluation of Sclerotium from a Medicinal Mushroom, *Lignosus cameronensis* (Cultivar): Preclinical Toxicology Studies

**DOI:** 10.3389/fphar.2017.00594

**Published:** 2017-09-01

**Authors:** Sook-Shien Lee, Nget-Hong Tan, Jayalakshmi Pailoor, Shin-Yee Fung

**Affiliations:** ^1^Department of Molecular Medicine, Faculty of Medicine, University of Malaya Kuala Lumpur, Malaysia; ^2^Department of Pathology, Faculty of Medicine, University of Malaya Kuala Lumpur, Malaysia

**Keywords:** *Lignosus cameronensis*, sclerotium, toxicity, hematological, histopathological

## Abstract

Twenty-eight days subacute toxicity studies performed in rats using sclerotial powder of *Lignosus cameronensis* cultivar was conducted to assess its safety for consumption prior to other scientific investigations on its medicinal benefits, nutraceutical or pharmaceutical application of the mushroom. The study was conducted at 250, 500, and 1000 mg/kg sclerotial powder of *L. cameronensis* cultivar (*n* = 5 for each respective dose, on both male and female groups) while control groups received only distilled water. At the end of the study (29th day), the animals were sacrificed followed by blood and organs collection for analysis. Subacute toxicity studies done shows that sclerotial powder of *L. cameronensis* cultivar at 250, 500, and 1000 mg/kg did not induce treatment related changes on behavioral patterns, gross physical appearance, growth pattern, body weight gain, values of hematological and clinical biochemical panels as well as histopathological findings on kidney, spleen, heart, lung and liver of the experimental rats. The no-observed-adverse-effect level dose for sclerotial powder of *L. cameronensis* cultivar in 28-days sub-acute toxicity study is determined to be 1000 mg/kg.

## Introduction

*Lignosus*, a genus of mushroom belonging to the Polyporaceae family, is mainly distributed in the tropical forest of South China, Thailand, Malaysia, Indonesia, Philippines, Papua New Guinea, Africa, and Australia (Ryvarden and Johansen, 1980; Cui et al., 2011; Tan et al., 2012). Six species of the genus (*Lignosus*) have been identified, namely *L. dimiticus, L. ekombitii, L. goetsii, L. sacer, L. hainanensis*, and *L. rhinocerotis* (Douanla-Meli and Langer, 2003; Cui et al., 2011; Tan et al., 2012). Two new species of *Lignosus* mushrooms have been collected from the tropical forest in the state of Pahang, Malaysia. They are identified and subsequently named as *L. tigris* and *L. cameronensis* (Tan et al., 2013). These two newly discovered *Lignosus* species and *L. rhinocerotis* were collectively known as Tiger Milk mushroom prior to their discovery and have now been successfully cultivated in the laboratory (Tan et al., 2013; Lau et al., 2015).

The sclerotium of *Lignosus* species are the part with medicinal value. In Peninsular Malaysia, the sclerotia of Tiger Milk mushroom are widely utilized by aborigines for the treatment of various ailments including cough, asthma, fever, food poisoning, cancer and as a general tonic (Lee et al., 2009). In China, the sclerotium is sold at a high price and is considered expensive among the other folk medicine in China. It has been used to treat gastric ulcers, chronic hepatitis and liver cancer (Wong and Cheung, 2009). Previous studies demonstrated that the sclerotia of *L. rhinocerotis* exhibit anti-proliferative, anti-oxidant, anti-microbial, anti-inflammatory, and immunomodulatory effect as well as stimulatory effect on neurite outgrowth (Lai et al., 2008; Wong et al., 2009, 2011; Eik et al., 2012; Lee et al., 2012; Mohanarji et al., 2012; Lau et al., 2013; Phan et al., 2013; Yap et al., 2013; Zaila et al., 2013; Lee et al., 2014). Non-digestible carbohydrates (NDCs) from the sclerotia may function as novel prebiotics (Gao et al., 2009). *L. tigris* was also reported to have anti-oxidant activity (Yap et al., 2014a; Kong et al., 2015).

Wide ethno-botanical usages of Tiger Milk mushrooms as well as several scientific reports on medicinal properties of *L. rhinocerotis* and *L. tigris* suggest that *L. cameronensis* likely has similar health benefit and has the potential to be developed into a nutraceutical. Prior to its successful cultivation, there were no studies done on the medicinal properties of *L. cameronensis* due to its limited supply from the wild. The advent of successful cultivation has enabled the exploration into its safety profile, medicinal and nutritional values.

In the present inquest, a 28-days sub-acute toxicity study on the sclerotial powder of *L. cameronensis* cultivar in rats was conducted to assess its safety for consumption prior to other scientific investigations on its medicinal benefits, nutraceutical or pharmaceutical application of the mushroom. In the experiments, male and female rats (*n* = 5 each) were randomly assigned into three treatment groups per gender and were orally administered with the sclerotial powder at three ascending doses while control groups received only distilled water. At the end of the study (29th day), the animals were sacrificed followed by blood and organs collection for analysis. The sub-acute toxicity study was conducted in accordance with the Organization of Economic Cooperation and Development guidelines (Organization for Economic Cooperation and Development [OECD], 2008).

## Materials and Methods

### Preparation of the Sclerotial Powder of *L. cameronensis* Cultivar

*Lignosus cameronensis* cultivar was obtained from Ligno Biotech Sdn. Bhd. (Selangor, Malaysia). The cultivation tissue of the cultivar originated from the wild type *L. cameronensis* which was collected from the tropical forest in Lata Iskandar (4°17.46′N 101°34.41′E), Pahang, Malaysia. The wild type mushroom was identified by internal transcribed spacer (ITS) regions of the ribosomal DNA via PCR technology (Yap et al., 2014b) [ITS-1 (5′-TT > GGCCCTT > CCCTT > CTGGCGG-3′) and ITS-2 (5′-GAGGCGCAGCCCTTCACTT-3′)]. Fifty milligram of clean sclerotial tissue was inoculated on potato dextrose agar (PDA) supplemented with 100 μg/L of streptomycin. Mycelial culture of 200 mg was sub-cultured into sterilized substrate containing 100 g of brown rice and 100 ml of distilled water in a polypropylene container. Spawn container were incubated in darkness at 24°C for 3 months. Sclerotia formed were then harvested, freeze dried and ground into powder by using 0.2 μm mesh filter. The sclerotial powder of the *Lignosus cameronensis* cultivar appears to be beige in color and light powdery in texture, similar to that of the wild type sclerotial powder.

### Animals

Male and female *Sprague Dawley* (SD) rats aged 5 weeks old were purchased from Sterling Ascent Sdn Bhd (Penang, Malaysia). Rats were housed in Animal Experimental Unit in University of Malaya under standard housing conditions of 12 h light/12 h darkness and 22 ± 2°C. Rats were given *ad libitum* access to standard chow diets and water. All animals were quarantined for at least 14 days prior to use. Transportation and care of the animals were performed in compliance with the relevant laws and experimental protocols reported in this study were approved by Institutional Animal Care and Use Committee, University of Malaya (UM IACUC-Ethics reference no.2015-180505/MOL/R/FSY).

### Oral Administration of *L. cameronensis* Cultivar Sclerotial Powder in Rats

Male (7 weeks old) and female (7–8 weeks old) *Sprague Dawley* (SD) rats were randomly divided into three treatment groups (250, 500, or 1000 mg/kg) and one control group per gender (one female and one male control group). Each experimental group consists of five animals. Sclerotial powder (at various dosages) was suspended in distilled water (10 ml/kg) and orally fed to the rats using oral gavage needles, once daily for 28 consecutive days. Control group received distilled water only during the study. Body weight of each rat was recorded daily and any changes in behavioral patterns and gross physical appearance were also observed every day. The dosages for treatment were selected based on OECD guidelines. Two lower doses, 250 mg/kg and 500 mg/kg were used to examine the presence or absence of dose related responses.

### Blood Analysis

On the final day of experimental study (day 28), rats were fasted for 18 h followed by anesthesia with ketamine (45 mg/kg) and xylazine (4.5 mg/kg). Blood collection was carried out by cardiac puncture. Examination of hematological and clinical biochemical parameters from blood sample were performed by using Advia 2120 Hematology System (Siemen, Germany) and Advia 2400 Chemistry System (Siemen, Germany), respectively. The parameters for hematological examination encompass red blood cell (RBC) count, hemoglobin concentration, packed cell volume (PCV), mean corpuscular volume (MCV), mean corpuscular hemoglobin (MCH), mean corpuscular hemoglobin concentration (MCHC), platelet count, white blood cell (WBC) count, neutrophil count, lymphocyte count, monocyte count, eosinophil count, basophil count and atypical lymphocyte count. Biochemical tests include glucose, urea, creatinine, calcium, inorganic phosphate, uric acid, sodium, potassium, chloride, total cholesterol, total protein, albumin, globulin, total bilirubin, alkaline phosphatase, serum glutamic oxaloacetic transaminase (SGOT or AST), serum glutamic pyruvic transaminase (SGPT or ALT) and gamma-glutamyl transpeptidase (GTT) (Lee et al., 2013).

### Histopathological Evaluations

Vital organs (kidney, spleen, heart, lung, and liver) were harvested and immersed in fixative (10% buffered formalin). The tissues were dehydrated by ethanol solution in serial concentrations, cleared with xylene and paraffin embedded. Paraffin blocks were sectioned with microtome into 5 μm thickness and each tissue section was stained with hematoxylin and eosin followed by microscopic examination by light microscope.

### Statistical Analysis

All data were presented as mean ± standard deviation. Statistical differences between the means of control and treatment groups were determined by One-way Analysis of Variance (ANOVA) followed by Dunnett’s t (two-sided) test. Dunnett T3 test was employed when homogeneity of variance was not fulfilled. Homogeneity of variances was calculated by using Levene statistics. Results were considered statistically significant when *p* < 0.05.

## Results

### General Observations

**FIGURE 1:**
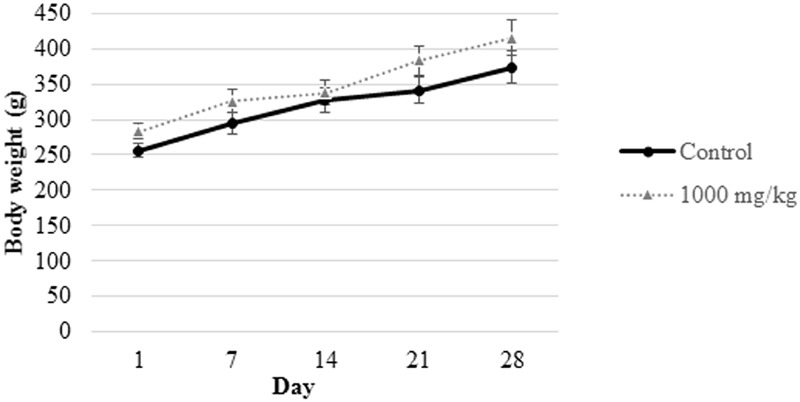
Body weight measurement of male rats from control group and those subjected to treatment with 1000 mg/kg *L. cameronensis* cultivar sclerotial powder. Body weight of rats are presented as mean ± SD (*n* = 5).

**FIGURE 2:**
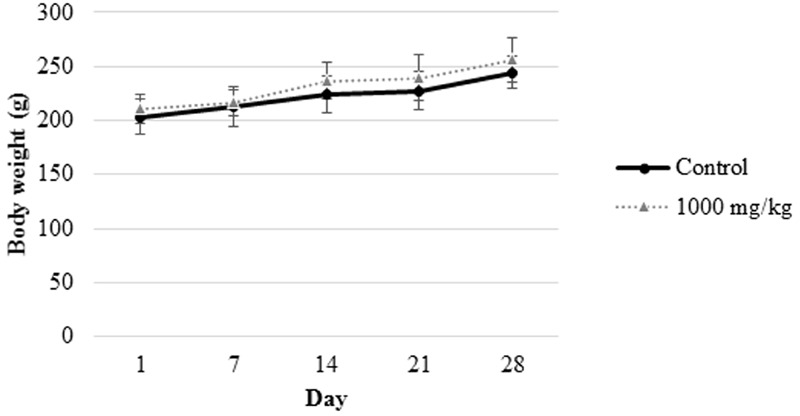
Body weight measurement of female rats from control group and those treated with 1000 mg/*L. cameronensis* cultivar sclerotial powder. Body weight of rats are presented as mean ± SD (*n* = 5).

Oral administration of the *L. cameronensis* cultivar sclerotial powder at various doses (250, 500, or 1000 mg/kg) did not induce alteration of fur and eye color. There was no occurrence of piloerection and diarrhea or impairment of locomotor activity in all the sclerotial administered rats. All animals survived. Growth rate for all treated rats were found to be similar to that of control rats from the same gender (**Figures 1, 2**). The net body weight gain of rats from all treatment groups were found to be comparable to the control rats (*p* > 0.05; **Table 1**).

**Table 1:** Body weight gain of male and female rats treated with varying doses of *L. cameronensis* cultivar sclerotial powder for 28 days.

Day	Body weight gain (g)							
	Male rats				Female rats			
	Control	250 mg/kg cultivar	500 mg/kg cultivar	1000 mg/kg cultivar	Control	250 mg/kg cultivar	500 mg/kg cultivar	1000 mg/kg cultivar
7	39.00 ± 6.78	37.20 ± 8.67	34.20 ± 8.70	42.40 ± 9.34	9.40 ± 6.91	10.80 ± 6.57	8.20 ± 8.79	5.60 ± 4.04
14	72.20 ± 8.64	67.20 ± 15.27	70.80 ± 16.04	54.00 ± 10.00	21.20 ± 6.94	14.00 ± 22.86	19.00 ± 11.96	26.00 ± 4.69
21	85.20 ± 11.39	101.60 ± 20.78	80.40 ± 11.99	99.40 ± 11.80	24.60 ± 5.41	35.20 ± 8.56	33.80 ± 11.71	28.60 ± 10.36
28	118.40 ± 16.15	130.80 ± 25.03	118.80 ± 21.06	131.60 ± 16.62	41.60 ± 7.89	44.80 ± 11.26	38.00 ± 14.87	45.40 ± 10.64

*Lignosus cameronensis cultivar sclerotial powder was orally fed to rats of both genders for 28 days consecutively. The values for body weight gain are presented as mean ± SD (*n* = 5/group/sex). There were no significantly differences in the net body weight gain of treated and control rats (*p* > 0.05).*

### Hematology

Hematological examinations of all male rats from the sclerotial administered groups (250, 500, and 1000 mg/kg) showed that the treatment did not induce significant changes in the values of RBC, hemoglobin, PCV, MCV, MCH, MCHC, platelet count, WBC, neutrophil, lymphocyte, monocyte, eosinophil, basophil and atypical lymphocyte (*p* > 0.05; **Table 2**). Other than MCV, MCHC and platelet counts, all of the hematological panels tested in all treatment groups of female rats were also found to be not significantly different from the control group (*p* > 0.05; **Table 3**). MCV levels in female groups treated with 500 mg/kg (53.40 ± 1.14 fL) and 1000 mg/kg (53.20 ± 0.84 fL) sclerotial powder were slightly lower than the control group (56.60 ± 1.34 fL). MCHC level in rats treated with 250 mg/kg (33.60 ± 0.55 g/dL) sclerotial powder was slightly lower than the control group (34.40 ± 0.55 g/dL). Platelet count in groups treated with 250 mg/kg (935.40 ± 45.37 ×10^9^/L), 500 mg/kg (923.20 ± 57.35 ×10^9^/L) and 1000 mg/kg (923.80 ± 45.23 ×10^9^/L) sclerotial powder were significantly different from the control group (795.80 ± 48.00 ×10^9^/L).

**Table 2:** Hematological values of male rats treated with three doses (250, 500, 1000 mg/kg) of *L. cameronensis* cultivar sclerotial powder for 28 days.

Treatment (mg/kg)	Control	250 mg/kg	500 mg/kg	1000 mg/kg
RBC (X10^12^/L)	8.34 ± 0.29	8.24 ± 0.39	8.24 ± 0.23	8.40 ± 0.44
Hemoglobin (g/dL)	16.16 ± 0.86	15.98 ± 0.82	15.66 ± 0.84	16.08 ± 0.83
PCV (%)	47.00 ± 2.24	47.00 ± 2.74	45.40 ± 2.30	47.20 ± 3.11
MCV (fL)	56.20 ± 1.92	57.00 ± 1.00	55.40 ± 1.34	56.40 ± 1.82
MCH (pg)	19.40 ± 0.55	19.40 ± 0.55	19.00 ± 0.71	19.20 ± 0.45
MCHC (g/dL)	34.60 ± 0.55	34.00 ± 0.00	34.40 ± 0.55	34.00 ± 0.00
Platelet count (X10^9^/L)	715.60 ± 42.02	773.20 ± 42.60	743.20 ± 58.51	744.40 ± 32.42
WBC (X10^9^/L)	5.22 ± 1.51	5.70 ± 1.37	6.10 ± 1.69	5.86 ± 2.06
Neutrophil (%)	8.40 ± 3.29	11.80 ± 1.79	11.20 ± 3.35	8.80 ± 2.39
Lymphocyte (%)	85.00 ± 2.92	84.20 ± 3.35	85.40 ± 2.51	84.20 ± 2.77
Monocyte (%)	3.40 ± 2.51	3.20 ± 1.10	6.00 ± 1.87	5.00 ± 1.87
Eosinophil (%)	0.00 ± 0.00	0.00 ± 0.00	0.40 ± 0.55	0.00 ± 0.00
Basophil (%)	0.00 ± 0.00	0.00 ± 0.00	0.00 ± 0.00	0.00 ± 0.00
Atypical lymphocyte (%)	0.00 ± 0.00	0.00 ± 0.00	0.00 ± 0.00	0.00 ± 0.00

*Values are reported as mean ± SD (*n* = 5/group). There were no statistically significant differences between treatment and control groups (*p* > 0.05).*

**Table 3:** Hematological values of female rats treated with three doses (250, 500, 1000 mg/kg) of *L. cameronensis* cultivar sclerotial powder for 28 days.

Treatment (mg/kg)	Control	250 mg/kg	500 mg/kg	1000 mg/kg
RBC (X10^12^/L)	7.76 ± 0.34	8.26 ± 0.83	8.50 ± 0.16	8.46 ± 0.23
Hemoglobin (g/dL)	15.14 ± 1.06	15.20 ± 1.33	16.30 ± 0.38	15.42 ± 0.36
PCV (%)	44.40 ± 2.61	46.00 ± 2.83	46.60 ± 0.89	45.00 ± 1.58
MCV (fL)	56.60 ± 1.34	55.60 ± 2.61	53.40 ± 1.14*	53.20 ± 0.84*
MCH (pg)	19.40 ± 0.55	18.60 ± 0.55	18.60 ± 0.55	18.60 ± 0.55
MCHC (g/dL)	34.40 ± 0.55	33.60 ± 0.55*	35.20 ± 0.45	34.40 ± 0.55
Platelet count (X10^9^/L)	795.80 ± 48.00	935.40 ± 45.37*	923.20 ± 57.35*	923.80 ± 45.23*
WBC (X10^9^/L)	4.42 ± 1.01	4.12 ± 1.49	5.00 ± 0.85	6.54 ± 2.19
Neutrophil (%)	8.20 ± 4.27	12.40 ± 4.45	7.40 ± 3.21	10.00 ± 3.08
Lymphocyte (%)	89.40 ± 4.39	81.80 ± 6.30	88.80 ± 3.27	83.80 ± 4.32
Monocyte (%)	5.80 ± 4.09	4.80 ± 1.30	4.60 ± 1.52	4.60 ± 2.41
Eosinophil (%)	0.40 ± 0.89	0.00 ± 0.00	0.00 ± 0.00	0.20 ± 0.45
Basophil (%)	0.00 ± 0.00	0.00 ± 0.00	0.00 ± 0.00	0.00 ± 0.00
Atypical lymphocyte (%)	0.00 ± 0.00	0.00 ± 0.00	0.00 ± 0.00	0.20 ± 0.45

*Values are reported as mean ± SD (*n* = 5/group). Asterisks (^∗^) are used to indicate significant differences to the control group [ANOVA, Dunnett’s t (two-sided) test, *p* < 0.05].*

### Clinical Biochemistry

**Tables 4, 5** shows the results of clinical biochemistry for male and female rats, respectively. After 28 days of feeding with the *L. cameronensis* cultivar sclerotial powder by oral gavage, serum biochemical parameters (serum glucose, urea, creatinine, calcium, inorganic phosphate, uric acid, sodium, potassium, chloride, total cholesterol, total protein, albumin, globulin, total bilirubin, alkaline phosphatase, SGOT, SGPT and GGT) of female rats from treatment groups were not markedly different from the control group (*p* > 0.05). In the male rats, most of the tested serum biochemical parameters showed insignificant differences between the treated and control rats, except for sodium and chloride levels. Sodium level in the male rats (146.60 ± 0.55 mmol/L) treated with 500 mg/kg sclerotial powder was slightly higher than in the control rats (144.00 ± 0.71 mmol/L, *p* < 0.05). Chloride level in male rats (102.40 ± 0.89 mmol/L) administered with 250 mg/kg sclerotial powder was slightly lower than the level in the control group (104.40 ± 0.55 mmol/L, *p* < 0.05).

**Table 4:** Clinical biochemistry of male rats treated with three doses (250, 500, 1000 mg/kg) of *L. cameronensis* cultivar sclerotial powder for 28 days.

Treatment (mg/kg)	Control	250 mg/kg	500 mg/kg	1000 mg/kg
Glucose (mmol/L)	7.02 ± 1.52	7.20 ± 1.24	8.00 ± 1.31	7.28 ± 0.99
Urea (mmol/L)	6.68 ± 0.78	6.28 ± 0.45	6.62 ± 0.46	5.94 ± 1.03
Creatinine (μmol/L)	17.60 ± 6.02	21.60 ± 4.67	20.60 ± 3.13	19.60 ± 2.88
Calcium (mmol/L)	2.38 ± 0.07	2.32 ± 0.03	2.36 ± 0.08	2.36 ± 0.18
Inorganic phosphate (mmol/L)	2.73 ± 0.30	2.63 ± 0.15	2.69 ± 0.06	2.68 ± 0.14
Uric acid (mmol/L)	0.05 ± 0.01	0.06 ± 0.01	0.05 ± 0.01	0.08 ± 0.06
Sodium (mmol/L)	144.00 ± 0.71	142.80 ± 0.45	146.60 ± 0.55*	144.40 ± 1.14
Potassium (mmol/L)	4.18 ± 0.28	4.14 ± 0.30	4.56 ± 0.23	4.80 ± 1.20
Chloride (mmol/L)	104.40 ± 0.55	102.40 ± 0.89*	102.60 ± 1.82	104.00 ± 1.22
Total cholesterol (mmol /L)	2.12 ± 0.36	2.36 ± 0.38	1.94 ± 0.34	2.00 ± 0.37
Total protein (g/L)	57.80 ± 3.03	60.20 ± 1.79	59.60 ± 3.21	59.40 ± 1.52
Albumin (g/L)	36.00 ± 2.35	37.40 ± 1.14	37.20 ± 2.05	36.80 ± 0.84
Globulin (g/L)	21.80 ± 0.84	22.80 ± 0.84	22.40 ± 1.34	22.60 ± 1.34
Total bilirubin (μmol/L)	1.00 ± 0.00	1.00 ± 0.00	1.00 ± 0.00	1.00 ± 0.00
Alkaline phosphatase (IU/L)	134.40 ± 19.50	176.20 ± 26.45	172.60 ± 39.18	161.20 ± 41.38
SGOT (AST) (IU/L)	140.80 ± 41.97	124.60 ± 8.32	107.80 ± 19.41	107.60 ± 30.00
SGPT (ALT) (IU/L)	58.80 ± 27.47	54.00 ± 7.18	51.60 ± 11.91	50.80 ± 7.53
GGT (IU/L)	0.00 ± 0.00	0.00 ± 0.00	0.20 ± 0.45	0.00 ± 0.00

*Values are reported as mean ± SD (*n* = 5/group). Asterisks (^∗^) are used to indicate significant difference to the control group [ANOVA, Dunnett’s t (two-sided) test, *p* < 0.05].*

**Table 5:** Clinical biochemistry of female rats treated with three doses (250, 500, 1000 mg/kg) of *L. cameronensis* cultivar sclerotial powder for 28 days.

Treatment (mg/kg)	Control	250 mg/kg	500 mg/kg	1000 mg/kg
Glucose (mmol/L)	6.92 ± 0.79	6.38 ± 0.40	6.76 ± 0.80	7.18 ± 0.81
Urea (mmol/L)	7.66 ± 1.15	5.62 ± 0.47	6.52 ± 1.34	6.72 ± 0.96
Creatinine (μmol/L)	26.60 ± 5.03	22.00 ± 1.58	20.00 ± 2.65	27.60 ± 5.86
Calcium (mmol/L)	2.50 ± 0.07	2.45 ± 0.07	2.41 ± 0.06	2.41 ± 0.06
Inorganic phosphate (mmol/L)	2.40 ± 0.26	2.25 ± 0.40	2.44 ± 0.15	2.31 ± 0.08
Uric acid (mmol/L)	0.06 ± 0.01	0.05 ± 0.01	0.06 ± 0.01	0.06 ± 0.01
Sodium (mmol/L)	144.80 ± 1.48	145.20 ± 2.17	145.80 ± 2.17	143.40 ± 1.52
Potassium (mmol/L)	3.94 ± 0.39	3.84 ± 0.27	3.92 ± 0.34	3.88 ± 0.15
Chloride (mmol/L)	103.40 ± 0.89	106.20 ± 3.27	103.40 ± 1.52	103.60 ± 1.52
Total cholesterol (mmol /L)	2.60 ± 0.21	2.20 ± 0.54	2.62 ± 0.49	2.42 ± 0.44
Total protein (g/L)	57.80 ± 4.87	59.00 ± 5.05	60.60 ± 2.79	61.40 ± 3.13
Albumin (g/L)	36.20 ± 3.42	36.80 ± 3.11	37.40 ± 1.82	39.00 ± 1.22
Globulin (g/L)	21.60 ± 1.82	22.20 ± 2.28	23.20 ± 1.30	22.40 ± 2.07
Total bilirubin (μmol/L)	0.60 ± 0.55	1.00 ± 0.00	1.00 ± 0.00	1.40 ± 0.55
Alkaline phosphatase (IU/L)	110.60 ± 6.02	88.40 ± 15.31	87.80 ± 23.66	119.20 ± 18.05
SGOT (AST) (IU/L)	115.00 ± 11.47	105.40 ± 9.07	103.40 ± 5.41	107.40 ± 16.92
SGPT (ALT) (IU/L)	45.00 ± 10.42	40.00 ± 8.86	48.20 ± 6.91	46.40 ± 6.88
GGT (IU/L)	0.00 ± 0.00	0.00 ± 0.00	0.00 ± 0.00	0.00 ± 0.00

*Values are expressed as mean ± SD (*n* = 5/dose). There were no statistically significant differences between treatment and control groups (*p* > 0.05).*

### Histopathological Examinations

**FIGURE 3:**
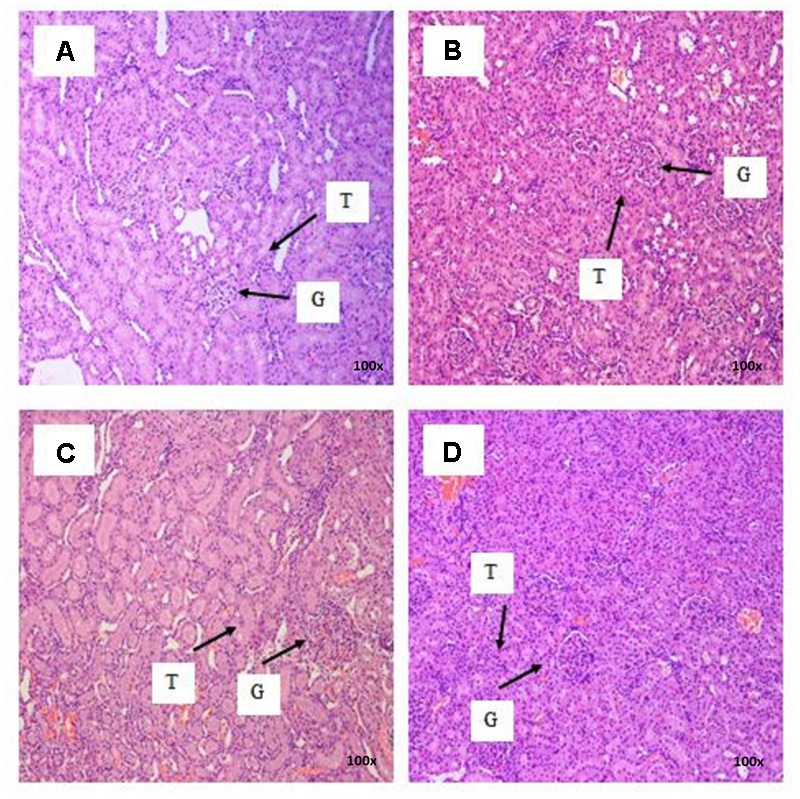
Photomicrograph of kidney sections from female rats treated with *L. cameronensis* cultivar sclerotial powder at different doses exhibited normal histological structure of glomerulus and tubules (x100 magnification; H&E stain). **(A)** control; **(B)** cultivar, 250 mg/kg; **(C)** cultivar, 500 mg/kg; **(D)** cultivar, 1000mg/kg. ‘G’ and ‘T’ indicate the position of glomerulus and tubule, respectively.

**FIGURE 4:**
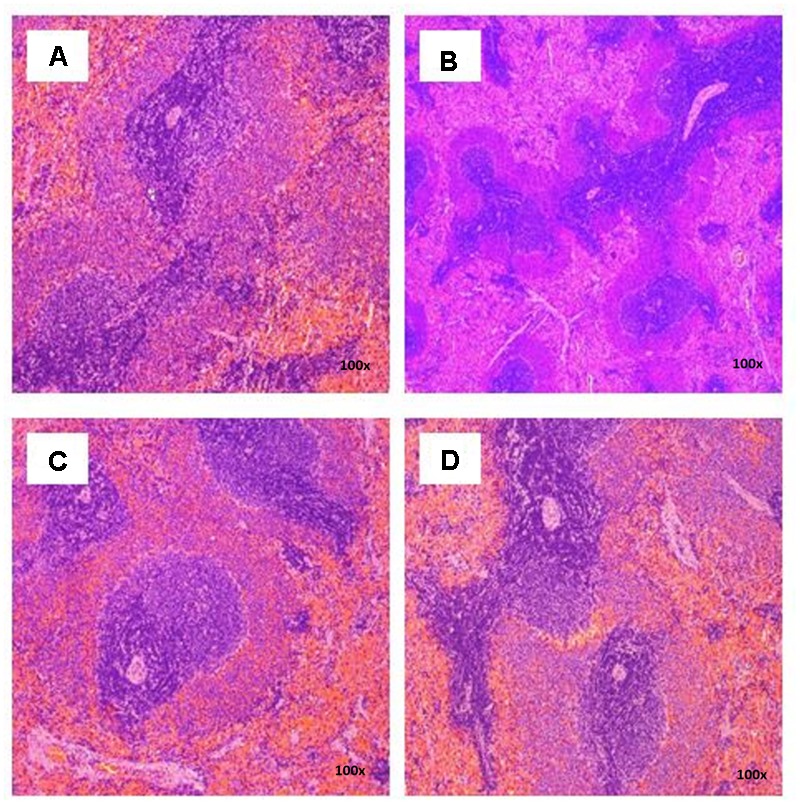
Photomicrograph of spleen sections from female rats treated with *L. cameronensis* cultivar sclerotial powder exhibited normal architecture on red and white pulp (x100 magnification; H&E stain). **(A)** control; **(B)** cultivar, 250 mg/kg; **(C)** cultivar, 500 mg/kg; **(D)** cultivar, 1000mg/kg.

**FIGURE 5:**
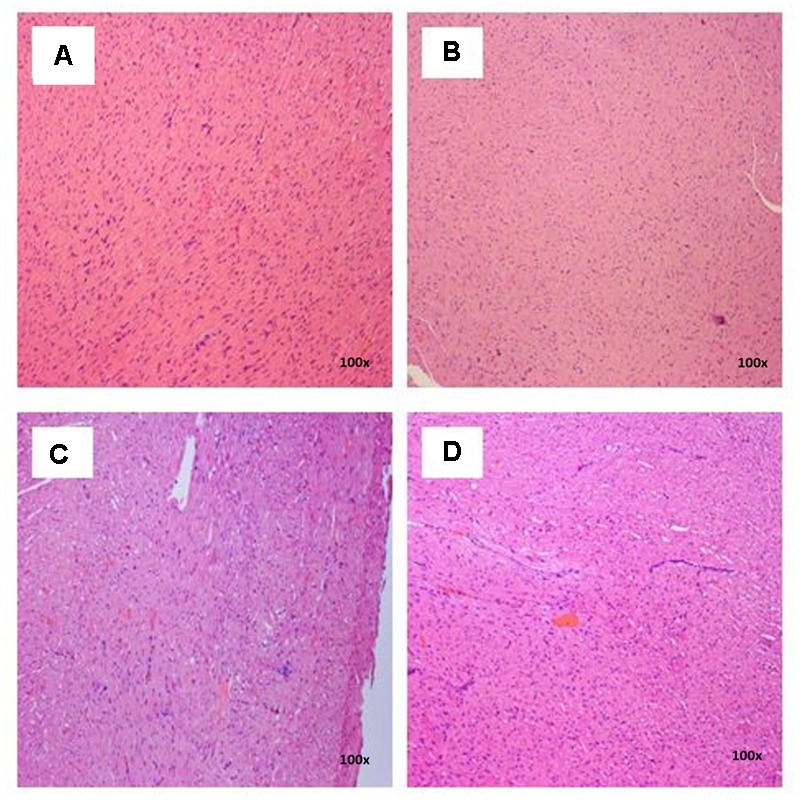
Photomicrograph of heart sections of female rats treated with *L. cameronensis* cultivar sclerotial powder exhibited normal appearance of myocardial fibers (x100 magnification; H&E stain). **(A)** control; **(B)** cultivar, 250 mg/kg; **(C)** cultivar, 500 mg/kg; **(D)** cultivar, 1000 mg/kg.

**FIGURE 6:**
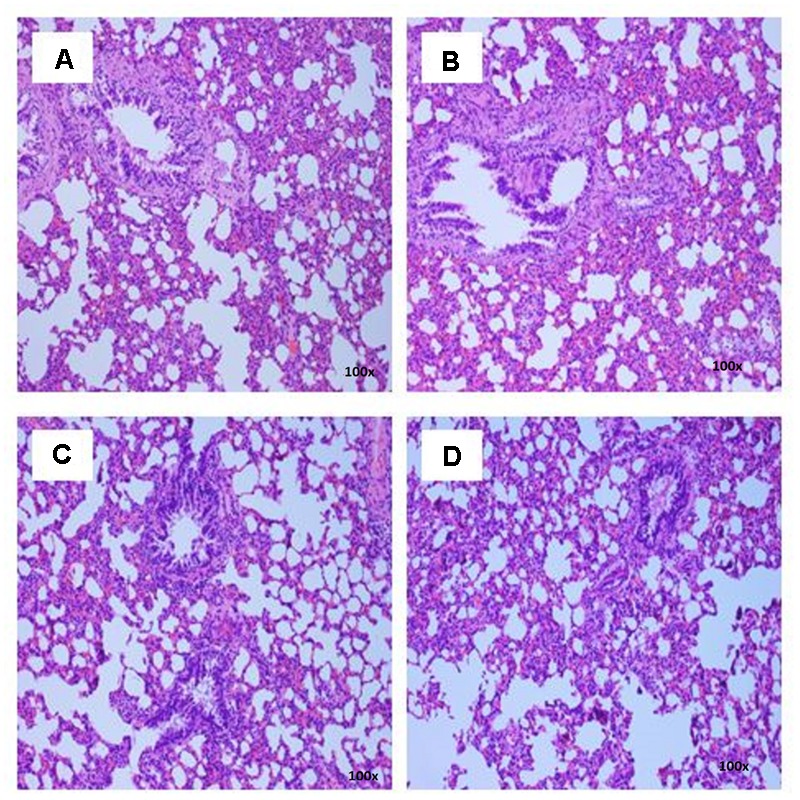
Photomicrograph of lung sections from female rats treated with sclerotial powder of *L. cameronensis* cultivar exhibited normal histological structure of bronchi and alveoli (x100 magnification; H&E stain). **(A)** control; **(B)** cultivar, 250 mg/kg; **(C)** cultivar, 500 mg/kg; **(D)** cultivar, 1000 mg/kg.

**FIGURE 7:**
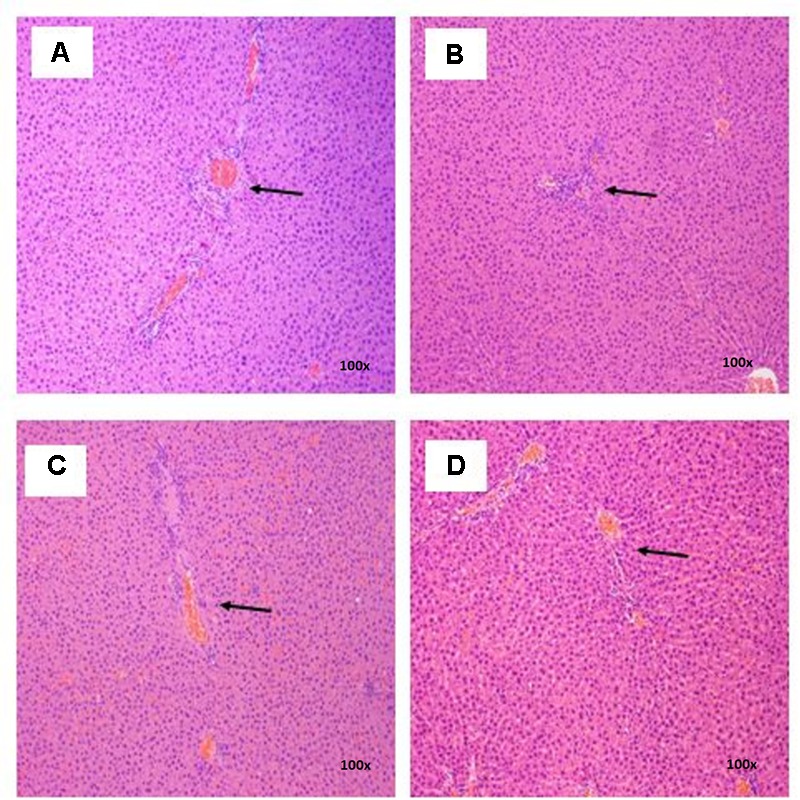
Photomicrograph of liver sections from female rats treated with sclerotial powder of *L. cameronensis* cultivar exhibited normal portal tracts and hepatocytes (x100 magnification; H&E stain). **(A)** control; **(B)** cultivar, 250 mg/kg; **(C)** cultivar, 500 mg/kg; **(D)** cultivar, 1000 mg/kg. Arrows show the position of portal tract.

**FIGURE 8:**
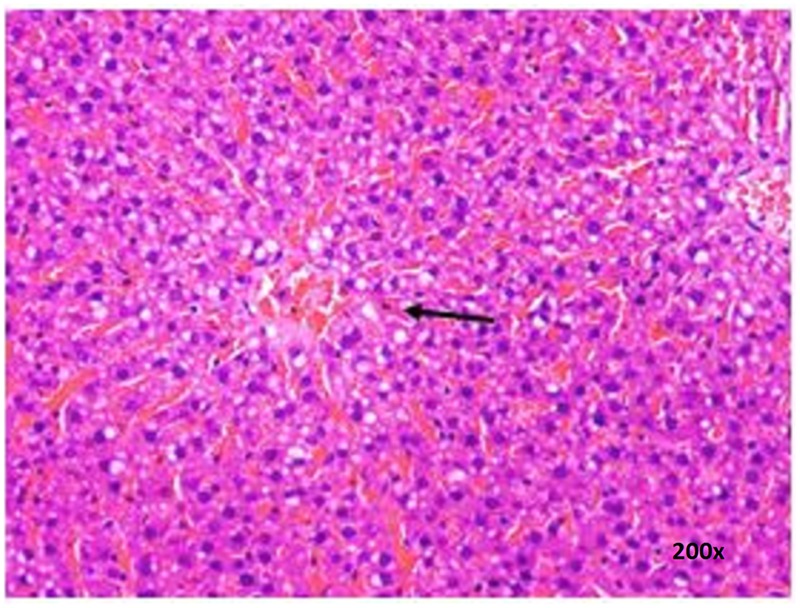
Photomicrograph of liver section of male rat displaying mild fatty change (x200 magnification; H&E stain) in control group. Arrow shows the area of fat contained in vacuolated cytoplasm of hepatocytes.

There were no treatment-related pathological changes in microscopic examination of kidney, spleen, heart, lung, and liver of female rats from both control and treatment groups, following oral feeding of *L. cameronensis* cultivar sclerotial powder for 28 consecutive days. Glomeruli and tubules were found to be normal in kidney tissues of control and treated groups (**Figure 3**). Histology of spleen (**Figure 4**), heart (**Figure 5**) and lung (**Figure 6**) of treated rats were also devoid of abnormality in which the spleen shows normal red and white pulps; the heart shows normal myocardial fibers; and the lung shows normal bronchi and alveoli without evidence of acute or chronic inflammation in the tissues. **Figure 7** shows normal portal tracts, central vein and hepatocytes in liver tissues of control and treatment groups. Neither necrosis of hepatocytes nor portal inflammation was observed. Similar histological observations of organs were noted in male rats, except for mild fatty change in liver tissue of control male rats (**Figure 8**). Similar findings were not observed in male rats that were administered *L. cameronensis* cultivar sclerotial powder at all the dosages in this sub-acute toxicity study.

## Discussion

In the present study, growth pattern (**Figures 1, 2**) and body weight gain (**Table 1**) of treated rats in both genders were found to be similar to that of the control group. This suggested that *L. cameronensis* cultivar sclerotial powder which was orally fed to the rats (up to 1000 mg/kg) for 28 consecutive days did not cause any harmful effect on the growth of the animals.

Hematopoietic system reflects physiological and pathological conditions in human and animal as it is one of the most susceptible targets for toxicant (Gautam and Goel, 2014). In general, our results show that the RBCs, white blood cells and platelet indices of rats administered with *L. cameronensis* cultivar sclerotial powder in three different doses (250, 500, and 1000 mg/kg) were not significantly different from the control group. Exceptions were noted in MCV, MCHC and platelet count of female rats. MCV levels of female rats administered with 500 and 1000 mg/kg of *L. cameronensis* cultivar sclerotial powder was slightly lower (*p* < 0.05) than that in the control group. The MCHC levels of female rats following administration of 250 mg/kg *L. cameronensis* cultivar sclerotial powder also showed slightly lower levels compared to the control group (*p* < 0.05). The minor variation in MCV and MCHC values were not of toxicological concern as they fell within normal reference range obtained from SD rats (Petterino and Argentino-Storino, 2006). The platelet counts in female rats administered with 250 mg/kg, 500 mg/kg, or 1000 mg/kg of the sclerotial powder showed significant difference from the control group. However, according to reference range reported by Patterino and Argentino-Storino, the values were considered normal for SD rats. Therefore, administration with *L. cameronensis* cultivar sclerotial powder for 28 days up to 1000 mg/kg did not induce any toxic effects on the blood cells and platelet of the rats.

Clinical biochemical examinations including liver function tests (total protein, albumin, globulin, total bilirubilin, alkaline phosphatase, SGOT, SGPT and GGT), and kidney function tests (urea, creatinine and uric acid levels) as well as serum electrolytes (calcium, inorganic phosphate, sodium, potassium, chloride), cholesterol and glucose levels of rats were carried out to further examine the safety of consumption of *L. cameronensis* cultivar sclerotial powder. In both groups of male and female rats administered with the sclerotial powder, the clinical biochemical parameters were not significantly different from the control group, except for sodium and chloride level of male rats. Sodium level of the male rats administered with 500 mg/kg *L. cameronensis* cultivar sclerotial powder was slightly higher (*p* < 0.05) than control. The chloride levels of male rats administered with 250 mg/kg *L. cameronensis* cultivar sclerotial powder was found to be slightly lower (*p* < 0.05) than the control group. Nevertheless, the values are within the normal ranges established by Patterino and Argentino-Storino for rats. Thus, the slight alterations in sodium and chloride levels are unlikely to be of toxicological concern.

Histological examinations of vital organs confirmed the findings obtained from clinical biochemistry. There were no observable histopathological changes in kidney, spleen, heart and lung of rats following oral administration of the *L. cameronensis* cultivar sclerotial powder up to 1000 mg/kg, for 28 consecutive days (**Figures 3–6**). Normal histological structures of liver were visualized in both male and female rats (**Figure 7**), except for mild fatty changes in the hepatocytes of the male control group (**Figure 8**). However, none of the treated groups exhibited fatty changes in hepatocytes.

## Conclusion

The results obtained from 28-days sub-acute toxicity study of the *L. cameronensis* cultivar sclerotial powder demonstrated that the consumption of the sclerotial powder is safe at dosage level up to 1000 mg/kg. The experimental rats did not show any changes in general observations, growth pattern and body weight gain. There were also no significant changes in the hematological, clinical biochemical parameters and histopathology of vital organs of rats. Therefore, the no-observed-adverse-effect level (NOAEL) dose of the *L. cameronensis* cultivar sclerotial powder in 28-day sub-acute toxicity study is up to 1000 mg/kg. This dosing level can serve as reference for long term repeated dose toxicity testing as well as assessment of reproductive toxicity, mutagenicity and genotoxicity of the *L. cameronensis* cultivar sclerotial powder.

## Author Contributions

S-YF and N-HT conceived and designed the study. S-SL performed the experiments, analyzed the data and drafted the manuscript. JP helped to perform and analyzed histopathological data. N-HT and S-YF helped to draft the manuscript.

## Conflict of Interest Statement

The authors declare that the research was conducted in the absence of any commercial or financial relationships that could be construed as a potential conflict of interest.
